# In the Twilight of Evidence: Is Bypass Surgery Still on the Table for Cardiac Allograft Vasculopathy?

**DOI:** 10.3390/jcm14010132

**Published:** 2024-12-29

**Authors:** Emyal Alyaydin, Andreas J. Flammer

**Affiliations:** Department of Cardiology, University Hospital Zurich, 8091 Zurich, Switzerland

**Keywords:** heart transplantation, cardiac allograft vasculopathy, coronary artery bypass graft surgery, surgical revascularization, retransplantation

## Abstract

**Background:** Cardiac allograft vasculopathy (CAV) is a major prognosis-limiting factor in patients undergoing orthotopic heart transplantation (HT). Due to the diffuse involvement of the coronary tree, CAV lesions are often not amenable to percutaneous coronary intervention (PCI), leaving coronary artery bypass grafting (CABG) and retransplantation as primary revascularization options. **Aim and Results**: The latest guidelines from the International Society for Heart and Lung Transplantation (ISHLT) recognize CABG as a viable option but with a downgraded strength of recommendation. The 2023 ISHLT guidelines now categorize CABG as a Class IIb recommendation (Level of Evidence: C) for highly selected CAV patients with anatomically suitable lesions, a downgrade from the Class IIa recommendation in the 2010 guidelines. This adjustment underscores the persisting reliance on limited, retrospective studies and the lack of substantial new data supporting CABG in CAV management. Our article examines the evidence collected since 2010 on this topic, highlighting key findings and assessing the role of CABG in contemporary transplant practice. This article calls for targeted investigations to better define the role of CABG as a therapeutic option, addressing the gaps in evidence for surgical revascularization in HT patients.

## 1. Introduction

Cardiac allograft vasculopathy (CAV) is a significant factor limiting long-term prognosis in patients undergoing orthotopic heart transplantation (HT). According to the 2019 report from the International Society for Heart and Lung Transplantation (ISHLT), CAV affects nearly 30% of HT recipients within five years and almost 50% within ten years post HT [1]. It is a leading cause of late graft failure and mortality, with particularly reduced survival rates when detected within the first three years after HT [1,2]. However, reporting rates may differ between centers, reflecting variations in assessment with invasive coronary angiography (ICA). In 2010, the ISHLT introduced a standardized nomenclature for CAV, aiming to unify classification and improve consistency in reporting. This nomenclature is based on ICA and echocardiographic findings and ranges from ISHLT CAV0 (not significant) to CAV3 (severe). Specifically, CAV0 indicates no angiographically detectable lesions, while CAV1, or mild CAV, includes cases with left main stenosis (LM) < 50% or primary vessel and branch stenosis < 70%, without associated allograft dysfunction. CAV2, or moderate disease, includes more severe stenoses (≥70%) in primary vessels or isolated branch stenosis, still without evidence of graft dysfunction or relevant LM stenosis (<50%). CAV3, or severe CAV, is defined by extensive disease with significant stenoses in multiple vessels or branches or by any CAV1 or CAV2 findings coupled with signs of allograft dysfunction, such as a left ventricular ejection fraction (LVEF) below 45% or restrictive physiology [3].

Despite the widespread use of ICA, intravascular ultrasound (IVUS) remains the gold standard for CAV detection [4,5]. IVUS is particularly valuable for its negative predictive value, helping to rule out mild disease or disease progression based on the evaluation of intimal thickening. However, the ISHLT does not recommend routine IVUS surveillance, as its benefits are primarily investigated in research settings rather than routine clinical practice [3].

Due to its progressive character and multifactorial genesis, the management of CAV presents substantial challenges. The diffuse nature of CAV lesions frequently renders them unamenable for percutaneous coronary intervention (PCI). Thus, coronary artery bypass grafting (CABG) and retransplantation (re-HT) are the common feasible treatment alternatives [6,7,8].

The ISHLT guidelines of 2010 and 2023 outline specific recommendations for CABG as a treatment modality for CAV. In the 2010 guidelines, CABG was recommended as a Class IIa option (Level of Evidence: C) for highly selected CAV patients with surgically accessible lesions. However, in 2023, the recommendation was downgraded to Class IIb, as there is still limited evidence and no substantial new data from the last decade [9,10].

Accordingly, the guideline recommendations regarding re-HT have evolved. The 2010 guidelines recommended re-HT for adult HT recipients with severe, untreatable CAV and accompanying symptoms of heart failure or ischemia (Class IIa, Level of Evidence: C). For asymptomatic patients with moderate to severe left ventricular dysfunction, re-HT was assigned a lower recommendation (Class IIb, Level of Evidence: C). In contrast, the 2023 guidelines now suggest that re-HT should be considered for both adults and children with severe CAV and allograft dysfunction if no contraindications exist (Class IIa, Level of Evidence: B) [9,10].

This article examines the evidence on CABG in HT accumulated since 2010, highlighting significant findings and assessing its role in current transplant care.

## 2. Evidence Since 2010

The evidence on CABG in HT patients reported over the past decade remains limited and is derived mainly from small studies with a low number of cases (Table 1).

The 2012 single-center observational study by Prada-Delgado et al. examined the outcomes of coronary revascularization procedures, including CABG, in HT recipients with moderate to severe CAV [11]. Of the 249 HT recipients, 43 had CAV (17 with CAV2 and 26 with CAV3). The mean age was 53.1 ± 12.5 years and the mean time from HT to revascularization was 4.6 ± 3.3 years. Of the forty-three patients with CAV, twelve (27.9%) underwent coronary revascularization, eight using PCI and four with CABG. The revascularization was performed for various reasons, including symptomatic ischemia (two patients with acute coronary syndrome (ACS)), high-risk asymptomatic CAV (six patients with lesions in the LM or proximal anterior descending artery or multivessel disease with left ventricular dysfunction), and ischemia identified on exercise echocardiography (four patients). The mean follow-up period for the study was 3.0 ± 2.4 years. Among the revascularized patients, 25% (three patients) experienced major adverse cardiovascular events (MACE), including death, acute coronary syndrome, or further cardiac complications. For those who underwent CABG, all procedures were successful with no perioperative mortality, reinforcing the viability of CABG in selected patients with severe CAV that is unsuitable for PCI [11].

The 2016 observational single-center study by Kuroda et al. explored the outcomes of coronary revascularization in patients with severe CAV. In this study, 96 HT recipients were screened for CAV, using ICA and IVUS, with a cut-off of >70% stenosis or a maximal intimal thickness (MIT) > 0.5 mm. Among these, 69 patients (71.9%) had CAV, with 5 patients diagnosed via ICA and 64 diagnosed using IVUS. All five of the patients with evidence of CAV on ICA underwent coronary revascularization, of which two patients underwent CABG (n = 2). Both had significant LM stenoses. The surgeries were successful, with no perioperative complications, and the follow-up angiography showed patent grafts (34 and 5 months after CABG) [12].

The 2018 national database analysis by Shah et al. focused on HT recipients who were hospitalized for ACS between 2007 and 2014, assessing trends in management, revascularization, and outcomes. Among 1621 patients with ACS, most presented with non-ST elevation myocardial infarction (NSTEMI) or unstable angina (UAP) (76%) rather than ST elevation myocardial infarction (STEMI) (24%). Of these, 36% patients underwent coronary revascularization, primarily using PCI. CABG was the preferred revascularization strategy in 14 patients. The study found significantly higher mortality in STEMI patients (28%) compared to those with NSTEMI/UAP (11%), as well as in those who did not undergo revascularization (19% vs. 7%). Patients with cardiogenic shock (CS), present in 14.5% of cases, had substantially higher mortality (39% vs. 10%) and more frequent use of mechanical circulatory support (MCS) than non-CS patients. MCS use in the CS group improved outcomes compared to those who did not receive it [13].

The 2022 multicenter observational study on cardiac surgery after heart transplantation (CASH) included data from 60 cardiac transplant centers, examining the indications, complications, and outcomes of cardiac surgery for valvular, coronary artery, or aortic disease following HT [14]. Of the 110 HT patients who were treated surgically, 16 underwent CABG. Among them, 14 (87.5%) were male, with a median age of 60.3 years and a median time from HT to surgery of 8.8 years. The indications for CABG included CAV in fourteen patients, iatrogenic LM dissection in one patient, and a right coronary artery stenosis not detected at the time of HT in one patient. Surgical access was primarily via re-sternotomy, with two patients undergoing minimally invasive direct coronary artery bypass (MIDCAB). The left internal mammary artery (LIMA) was used in 81.3% of cases and saphenous vein grafts in 50%. Postoperative outcomes revealed a median ICU stay of 2.5 days and an in-hospital stay of 12.5 days. The 1-year survival rate was 93.8%, with one death related to the surgery (on postoperative day 59 after iatrogenic LM dissection). Half of the patients had a progressive CAV and underwent PCI with drug-eluting stents, while one patient required a re-HT in the follow-up. The study concluded that CABG in HT recipients is a viable option for managing CAV, with favorable long-term outcomes in selected cases [14].

## 3. Discussion

The presented evidence demonstrates that CABG can be successfully implemented in the treatment of highly selected HT recipients with severe CAV. However, much of this information is derived from single-center observational studies with limited case numbers. In the studies by Prada-Delgado et al. and Kuroda et al. only a small number of patients were treated with CABG (four and two, respectively) [11,12]. The analysis by Shah et al. offers a broader perspective but primarily focuses on revascularization in HT recipients hospitalized with ACS rather than on CABG outcomes specifically [13]. While this study suggests that revascularization may improve outcomes in patients with ACS, its design does not allow for a detailed analysis of the role of CABG, particularly in non-ACS contexts or over long-term follow-up periods. The 2022 multicenter observational study on CASH adds valuable insights to the limited evidence base on CABG in HT recipients [14]. In line with previous findings, the study reported favorable short-term outcomes, with a 1-year survival rate of 93.8% and no significant perioperative mortality. These limitations underscore a key issue: despite the clinical relevance of CABG for severe CAV in HT recipients, the current evidence is insufficient to define short- or long-term outcomes for this patient population. However, none of the studies reported poorer perioperative outcomes among CABG patients, with no perioperative mortality documented in the small samples of HT recipients who underwent CABG [11,12]. These findings suggest that, in carefully selected cases, CABG may still be a viable revascularization option in patients with advanced CAV. However, the CASH study highlights the potential for the progression of CAV, with half of the patients requiring subsequent PCI and one patient requiring re-HT, reinforcing the complexity of CAV management [14].

A previous comprehensive review and meta-analysis by El-Andari et al., published in 2022, provided an extensive analysis of revascularization strategies in CAV [15]. The article included 24 studies, with only 4 reporting on CABG. Of these, three were published after 2010 (Prada-Delgado et al., Shah et al., and Kuroda et al.) and one before 2010 (Bhama et al.) [7,11,12,13]. In contrast, PCI was the preferred treatment strategy in all other cases. Considering the reported complications, such as major bleeding and higher rates of restenosis and revascularization in PCI patients, further research into the long-term outcomes of CABG in CAV patients is necessary to guide clinical decision making.

Nevertheless, HT recipients undergoing CABG or re-HT represent a population at inherently high perioperative risk. Complications may arise due to immunosuppressive therapy, delayed wound healing and increased susceptibility to infections, and potential comorbidities, such as impaired renal or ventricular function. Given the high-risk profile of HT recipients, an individualized risk assessment and optimal perioperative management are essential when considering surgical therapy [16,17].

Future research should focus primarily on the prevention of CAV and also address the gaps in evidence regarding treatment alternatives by prioritizing multicenter studies, including examining CABG outcomes in HT recipients more comprehensively. These combined efforts will help optimize the prognosis of HT recipients. There is a need for robust evidence regarding the safety, efficacy, and long-term benefits of CABG in HT recipients facing complex coronary pathologies.

## 4. Conclusions

The current guidelines assign a Class IIb recommendation with Level of Evidence C for CABG in HT recipients with severe CAV, reflecting the limited available data. CABG may be a viable and potentially safe option for selected HT recipients with advanced CAV. There is a need for multicenter studies that comprehensively assess the safety, efficacy, and long-term outcomes of CABG in this context. Robust evidence would provide an invaluable foundation for refining treatment strategies and improving long-term prognosis in this unique patient population.

## 5. Limitations

This review has several limitations including the limited scope of existing studies, most of which are single-center observational analyses with small numbers of CABG cases. Additionally, current research lacks dedicated focus on CABG-specific outcomes in HT recipients beyond the perioperative period. HT recipients generally face substantial perioperative risks due to factors such as immunosuppression, delayed wound healing, and comorbidities, which were not comprehensively addressed in these studies. Future multicenter efforts and registries are essential to evaluate CABG in HT recipients systematically, providing robust data on long-term outcomes and helping guide treatment strategies in patients facing complex coronary pathologies post HT.

## Figures and Tables

**Table 1:** Studies on CABG in HT since 2010.

Studies Since 2010	Study Type	CABG/Patients with CAV *n* (%)	Male n (%)	Mean Age Years	Time HT–CABG Months/Years	CVRF *n* (%)	Follow-Up After CABG Months/Years	MACE n (%)
Prada-Delgado et al., 2012 [11]	Observational study	4/12 (33.3)	NR	53.1	4.6 ± 3.3 years	NR	3.0 ± 2.4 years	0
Kuroda et al., 2016 [12]	Observational study	2/5 (40.0)	4 (80.0)	49.5	147.4 ± 125.0 months	AH, 3 (60.0) DM, 3 (60.0) DL, 3 (60.0)	19.5 ± 20.5 months	0
Shah et al., 2018 [13]	National database analysis	14/576 (2.4)	401 (69.6) *	54.3 *	NR	AH, 140 (24.3) DM, 149 (25.9) DL, 272 (47.2)	Postoperative in-hospital follow-up	In-hospital mortality (CABG + PCI) 39 (6.8)
Gökler et al., 2022 [14]	Multicenter observational study	16/14 (N.A.) **	14 (87.5)	60.3	8.8 years	DM 4 (25.0) ***	In-hospital follow-up	In-hospital mortality—1 (6.3)

HT—heart transplantation, PCI—percutaneous coronary intervention, CABG—coronary artery bypass graft surgery, LVEF—left ventricular ejection fraction, CVRF—cardiovascular risk factors, MACE—major adverse cardiovascular events, AH—arterial hypertension, DM—diabetes mellitus, DL—dyslipidaemia, NR—not reported, ACS—acute coronary syndrome. Data are presented as mean ± SD or number (percentage). * Data regarding all patients with CAV (including those who were not treated or were treated with PCI). ** A total of 16 patients treated with CABG—of these, 14 patients with CAV, 1 patient with left mainstem dissection and 1 patient with stenosis of the right coronary artery. *** Only DM reported.
